# Sequencing an F1 hybrid of *Silurus asotus* and *S. meridionalis* enabled the assembly of high-quality parental genomes

**DOI:** 10.1038/s41598-021-93257-x

**Published:** 2021-07-05

**Authors:** Weitao Chen, Ming Zou, Yuefei Li, Shuli Zhu, Xinhui Li, Jie Li

**Affiliations:** 1 Pearl River Fisheries Research Institute, Chinese Academy of Fishery Science, Guangzhou, 510380 China; 2 Experimental Station for Scientific Observation on Fishery Resources and Environment in the Middle and Lower Reaches of Pearl River, Zhaoqing, China; 3 Institute of Zoology, Chinese Academy of Sciences, Beijing, China

**Keywords:** Evolution, Zoology

## Abstract

Genome complexity such as heterozygosity may heavily influence its de novo assembly. Sequencing somatic cells of the F1 hybrids harboring two sets of genetic materials from both of the paternal and maternal species may avoid alleles discrimination during assembly. However, the feasibility of this strategy needs further assessments. We sequenced and assembled the genome of an F1 hybrid between *Silurus asotus* and *S. meridionalis* using the SequelII platform and Hi-C scaffolding technologies. More than 300 Gb raw data were generated, and the final assembly obtained 2344 scaffolds composed of 3017 contigs. The N50 length of scaffolds and contigs was 28.55 Mb and 7.49 Mb, respectively. Based on the mapping results of short reads generated for the paternal and maternal species, each of the 29 chromosomes originating from *S. asotus* and *S. meridionalis* was recognized. We recovered nearly 94% and 96% of the total length of *S. asotus* and *S. meridionalis*. BUSCO assessments and mapping analyses suggested that both genomes had high completeness and accuracy. Further analyses demonstrated the high collinearity between *S. asotus*, *S. meridionalis*, and the related *Pelteobagrus fulvidraco*. Comparison of the two genomes with that assembled only using the short reads from non-hybrid parental species detected a small portion of sequences that may be incorrectly assigned to the different species. We supposed that at least part of these situations may have resulted from mitotic recombination. The strategy of sequencing the F1 hybrid genome can recover the vast majority of the parental genomes and may improve the assembly of complex genomes.

## Introduction

Heterozygosity strongly influences the de novo assembly of eukaryotic genomes. High heterozygosity may result in poor continuousness, with a number of redundant and short sequences^1^. For a de novo assembly, it is best to select a sample with low heterozygosity, or getting a sample from an inbred line, but this is usually difficult for some species. Sequencing a hybrid F1 genome of two related species may be useful for obtaining more continuous assemblies, since somatic cells harbor two haploid copies of the parental species. These should allow easier generation of a more continuous assembly^2^.

However, some features of the hybrid genome may hinder obtaining the “real genome” of the parental species. The first feature is “genome shocking,” such as the disrupting of gene regulation and transposable element (RE) silencing. These may result from the confusion of DNA methylation and histone methylation in the somatic cells of hybrids^3,4^. The extensive activation of REs may result in their propagations in the hybrid genome, causing insertions and deletions across the genome^5^. Another issue is the possible mitotic recombination between parental genomes in somatic cells of the hybrid. This may result in chimeric sequences of the two parental species, although the frequency of occurrence may be rare and occur more in aged adults^6,7^. However, these impacts may be attenuated when many somatic cells were sequenced, and the assembly process may reconcile their differences and construct consensus sequences.

*Silurus asotus* and *S. meridionalis* diverged more than 15 million years ago^8^. Both of the two species are important cultured fish species in China. *S. asotus* has a superior taste, but *S. meridionalis* grows rapidly. Hybrids generated using *S. asotus* as father and *S. meridionalis* as mother have the best qualities of both parents. They exhibit rapid growth like *S. meridionalis* and preferred taste like *S. asotus*.

To test the feasibility of sequencing and assembling genome sequences of the two parental species in the somatic cells of their hybrids, we downloaded the whole genome sequencing raw reads of *S. asotus* and *S. meridionalis* from public databases, and compared these with the entire genome sequence of an F1 hybrid individual generated in the present study. We demonstrated that the strategy of sequencing the hybrid genome can obtain the two parental genomes with high continuousness and accuracy. However, a small portion of chimeric sequences that may have resulted from mitotic recombination may also be obtained, and this should be attenuated using species with more divergent genome sequences. Thus, by appropriate choice of the two parental species, this strategy should be useful when sequencing species with high heterozygosity, and even polyploidy species, for which the continuousness of their assembly has always been hampered by the complexity of the genome^9^. Or, the newly generated assembly could be used as an accurate reference genome to assist locating and orientation of the contigs assembled for the non-hybrid parental individuals, as implemented in software such as MUMmer4 and RaGOO^10,11^.

## Materials and methods

### Sampling and sequencing

A living sample of F1 offspring generated using *S. asotus* hybrid with *S. meridionalis* was purchased in a fish market located in Guangzhou, China. The maternal role of *S. meridionalis* was corroborated by a COI fragment. Tissues of white muscle, intestines, skin, kidney, gill, and spleen were dissected after injecting with MS-222 (MS-222, TMS, tricaine methanesulfonate), and were immediately frozen in liquid nitrogen until use. Total DNA was extracted from white muscle using the classic Phenol–chloroform method and was used to prepare libraries for whole genome sequencing. For sequencing using the PacBio SequelII platform with the continuous long read sequencing (CLR) model, library was constructed as described for *Lycorma delicatula*^12^. For sequencing using the Illumina HiSeq 2000 platform with the paired-end model, library was prepared using the Illumina TruSeq DNA library preparation kit (Illumina) according to manufacturer instructions. The insert size for the library was 350 bp. White muscle was also used to construct the high-throughput chromosome conformation capture (Hi-C) library by adopting the protocol used for *Jatropha curcas*^13^. The library was sequenced with the HiSeq 2000 platform with paired-end model. Total RNAs were extracted from five tissues of intestines, skin, kidney, gill, and spleen using TRIzol (Invitrogen), and were used to construct libraries, which were then sequenced using the HiSeq 2000 platform.

### De novo assembly of the genome

The Illumina short reads generated for the hybrid genome were used to survey its total size, heterozygosity, and repeat contents. The raw reads were quality controlled using fastp v0.20.1with default settings^14^, and were then feed into kmerfreq^15^ to survey the genome. For comparison, genomic short reads for the two non-hybrid parental species deposit in NCBI SRA database (https://www.ncbi.nlm.nih.gov/sra) were downloaded and used to survey the two parental genomes following the same pipeline as aforementioned (Supplemental Table S1). Heterozygosity of the genome were estimated using GenomeScope1.0^16^. After that, the PacBio long reads were used to de novo assemble the hybrid genome using Falcon v0.2.2 with the parameters “--length_cutoff=21000 --length_cutoff_pr=20000 --max_diff 100 --max_cov 80 --min_cov 2 --bestn 10”^17^. Arrow v2.3.3 was used to improve the assembly, based on the alignments of raw reads mapped back to the assembly using pbmm2 with default settings (https://github.com/PacificBiosciences). Pilon v1.23^18^ was then used to polish the assembly iteratively for three times, based on the alignments of short reads mapped back to the assembly using BWA-MEM v0.7.17 with default settings^19^. For scaffolding, the Hi-C reads were quality controlled using fastp v0.20.1 with default settings, and were then mapped back to the assembly to generate the contact matrix using Juicer v1.5^20^. Based on the matrix, 3d-dna^21^ was then used to scaffold the contigs using default settings, which were then adjusted manually using Juicebox v1.22^22^.

### Division and assessments of the genome

The downloaded short reads were mapped back to the new assembly using BWA-MEM v0.7.17 with default settings^19^. The alignments were then merged using Samtools v1.10^23^ for each of the two parental species, *S. asotus* and *S. meridionalis*. The coverage and mean depth of total reads for each of the two parental species were estimated using Samtools v1.10 and were used to divide the two parental chromosomes of the hybrid individual. After that, all of the short reads were mapped back to the divided chromosomes for each of the species, and the mapping rate was estimated. The insert lengths of the alignments were also estimated for the libraries with insert sizes less than 1 kb. Completeness of the two divided genomes was estimated using Benchmarking Universal Single-Copy Orthologs (BUSCO v3.0.2)^24^ with the 3640 genes included in actinopterygii_odb10 as the reference. For comparison, the estimation was also performed for the de novo assembled hybrid genome.

### Comparative genomics analyses of the divided genomes

The collinearity analyses between chromosomes of the two divided genomes, and between each of the divided genomes with the relative species *Pelteobagrus fulvidraco* (yellow catfish) reported before^25^ were performed, respectively. Briefly, possible homologous regions between the two genomes subject to comparison were identified using MUMmer4 with default settings^11^, and were presented using Circos v0.69-8^26^. To identify possible recombination between chromosomes of different species in the hybrid somatic cells, we compared the genomes of the two non-hybrid parental species with that of the hybrid individual. Genome sequences for each of the two parental species were assembled using SOAPdenovo2^27^ with default settings based on the quality controlled short reads downloaded from SRA, and were concatenated and compared to the de novo assembled genome for the hybrid individual using MUMmer4 with default settings^11^. The summary of the comparison and the coordinates of 1-to-1 alignment blocks were all generated with dnadiff included in MUMmer4.

### Annotations of the divided genomes

Annotations of REs and gene-models residing in the divided genomes were performed separately for each of the parental species. REs residing in the genome were identified using Extensive *de-novo* TE Annotator (EDTA v1.8.3), which integrates de novo identified LTRs using LTR_retriever^28^, and other de novo identified REs using RepeatModeler^29^, and the known REs deposited in Repbase^30^ to generate a non-redundant library, which was used to query the target genome to identify all REs residing in the genome using RepeatMasker^31^. Gene-models identifications included three strategies: homolog-based, RNA-seq-based, and de novo methods. For homolog-based annotations, protein sequences of the related species *P. fulvidraco*, *Pangasianodon hypophthalmus*, *Ictalurus punctatus*, *Bagarius yarrelli*, and *Danio rerio* were downloaded from public databases and were queried against the target genome using tblastn^32^. Homologous regions were then extracted using GenBlastA^33^ based on the alignments, and the possible coding sequences were predicted based on the comparison of protein sequences and the extracted homologous regions using genewise v2.4.0 with default settings^34^. For the second strategy, Illumina short reads for each of the five tissues were quality controlled using fastp v0.20.1^14^, and were de novo assembled using Trinity v2.11.0 with the genome-guided model^35^. Then the sequences were fed into the Program to Assemble Spliced Alignments (PASA v2.4.1)^36^ to generate the most plausible transcripts, which was used to predict the possible gene models with Transcoder v0.9.1^37^. The predicted gene models were subsequently used to train models for Augustus v3.3.2, Glimmer v3.02, and SNAP^38–40^, and possible coding regions were predicted based on the models. Finally, all the gene models were combined and used to generate a most probable gene set for the target species using EVM v1.1.1^36^, with different weights assigned to different strategies.

## Results

### Raw data

More than 300 Gb of raw data was generated for the hybrid individual in the present study (Supplemental Table S2). Specifically, more than 8 million long reads totaling 126.38 Gb, more than 773 million short reads totaling 115.97 Gb, and more than 712 million short reads totaling 106.92 Gb were generated for the de novo assembly, survey analyses, and Hi-C scaffolding, respectively. More than 6 Gb raw data were generated for each of the transcriptomes of intestines, skin, kidney, gill, and spleen. Genomic short reads for the two non-hybrid parental species were downloaded from NCBI SRA database (https://www.ncbi.nlm.nih.gov/sra, Supplemental Table S1).

### Genome assembly

**Figure 1:**
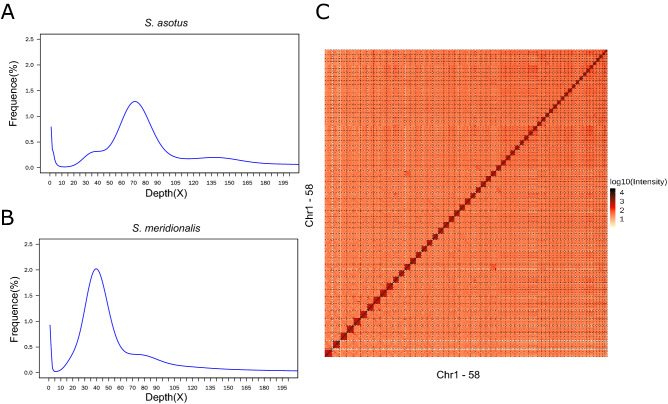
Assembly features of the hybrid genome. (A,**B**) 17-mer distributions deduced using short reads from non-hybrid individuals for the parental species *S. asotus* and *S. meridionalis*, respectively. (**C**) The heatmap of interactions between any two regions across the chromosomes residing in the somatic cells of the hybrid individual estimated based on the Hi-C sequencing data.

Assembly of the hybrid genome was performed based on the data generated. Based on the genomic short reads the size of the hybrid genome was estimated to be 1.50 Gb (Table 1; Supplemental Figure S1), nearly equal to the sum of the genome sizes of the two non-hybrid parental species estimated using genomic short reads downloaded from SRA, which were 791.52 Mb for *S. asotus*, and 780.35 Mb for *S. meridionalis*, respectively (Table 1; Fig. 1). Moreover, heterozygosity of the hybrid genome is extremely high (~ 3.67%), suggesting the estimated genome size should be diploid. Given the estimated genome size for the hybrid individual, de novo assembly based on the long-reads generated 3017 contigs, with a contig L50 and N50 of 63 and 7.49 Mb, respectively (Table 1). Hi-C scaffolding located and oriented 731 contigs onto 58 scaffolds confidently (Fig. 1), with a scaffold L50 and N50 of 24 and 28.55 Mb, respectively. The total length of the hybrid genome, and the divided *S. asotus* and *S. meridionalis* genome are approximate to 1.54 Gb, 744.12 Mb, and 748.79 Mb, respectively (Table 1). Precisely, the total length of the anchored contigs accounted for 96.66% of the assembly, and thus the 58 scaffolds may represent the 58 chromosomes of the hybrid individual and the assembly is nearly chromosomal^41^. BUSCO assessments suggested that about 80.50% of the 3640 near-universal single-copy orthologs in Actinopterygii are duplicated in the hybrid genome, and another 17% are single-copy, and about 0.20% are fragmented and 2.30% are missing (Supplemental Figure S2).

**Table 1:** Summary of indexes of the de novo assembly of the *S. asotus* × *S. meridionalis* hybrid genome.

Indexes	*S. asotus* × *S. meridionalis*	*S. asotus*	*S. meridionalis*
Estimated genome size	1.50 Gb	791.52 Mb	780.35 Mb
percent assembled	102.6%	94.0%	96.0%
Scaffold number	2344	29	29
Total scaffold length	1.54 Gb	744.12 Mb	748.79 Mb
Scaffold L50	24	12	12
Scaffold N50 length	28.55 Mb	28.82 Mb	28.19 Mb
Contig number	3017	373	358
Total contig length	1.54 Gb	744.09 Mb	748.75 Mb
Contig L50	63	38	24
Contig N50 length	7.49 Mb	6.75 Mb	9.78 Mb

### Division of the hybrid genome

**Figure 2:**
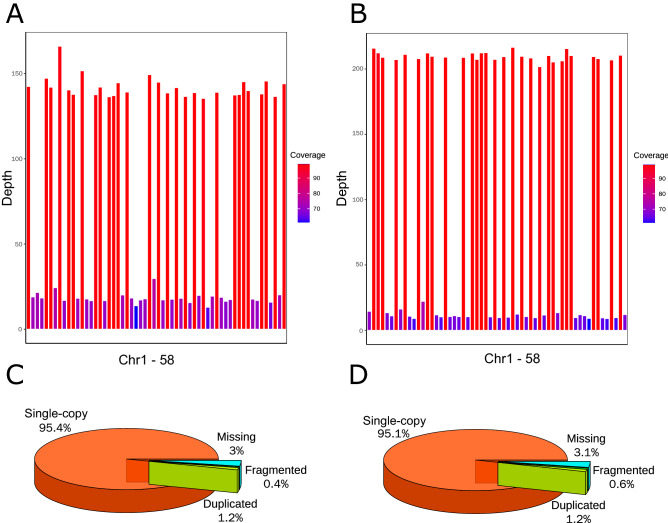
Assessments of the de novo assembly. (**A**, **B**) The coverages and meandepths of genomic short reads from non-hybrid parents mapped to the de novo assembly for *S. asotus* and *S. meridionalis*, respectively. (**C**,**D**) are the BUSCO assessment results for the *S. asotus* and *S. meridionalis* genomes divided from the hybrid genome, respectively.

**Figure 3:**
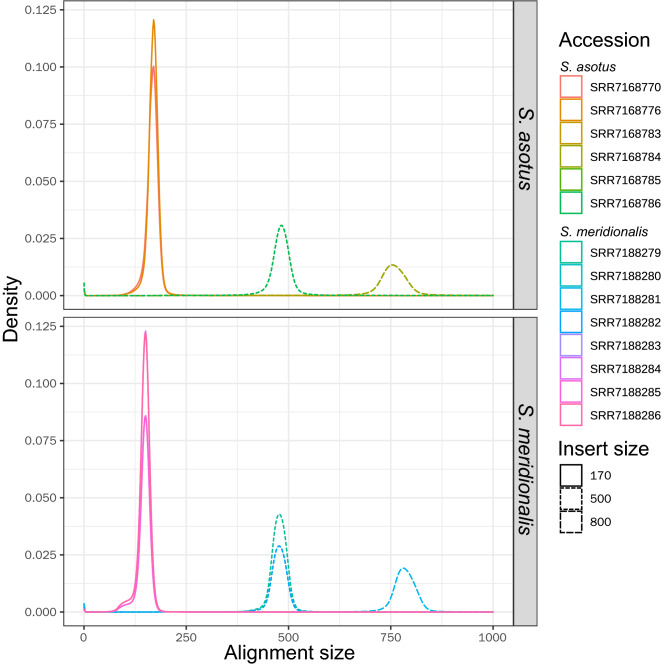
The insert length distribution estimated using the genome sequences divided from the hybrid genome as reference for each of the libraries constructed for the non-hybrid parental species *S. asotus* and *S. meridionalis*.

The assembly of the hybrid genome was divided and assessed separately. The genomic short reads for each of the non-hybrid individuals of the two parental species were mapped back to the assembly, and the chromosomes from *S. asotus* and *S. meridionalis* were recognized based on the mapping depth and coverage as defined in Samtools (Fig. 2). Using samtools, the proportion of covered bases and the mean depth of coverage were summarized and termed coverage and meandepth, respectively. When the short reads of *S. asotus* were mapped back, nearly all of the regions of 29 chromosomes are highly covered, but for the other 29 chromosomes, the depth decreased sharply, and the coverage were declined, too. Thus, we were able to identify the chromosomes from the two species. When the short reads of *S. meridionalis*, the situation was similar (Fig. 2). The assembly sizes for *S. asotus* and *S. meridionalis* were 744.12 Mb and 748.79 Mb, representing 94.0% and 96.0% of the estimated genome sizes, respectively (Table 1). BUSCO assessments suggested that more than 95% of the genes were single copy and complete, and less than 1% were missing for each of the two species (Fig. 2). The contig N50 lengths were 6.75 Mb and 9.78 Mb for *S. asotus* and *S. meridionalis*, respectively. The high completeness and continuity, as well as the high mapping rates of the genomic short-reads from the parental species (Supplemental Table S1), suggest the high quality of the two assemblies. It is also worth noting that the insert sizes of the mapping results were similar to the insert fragment lengths when we scrutinized the alignments of libraries with insert fragments shorter than 1 kb (Fig. 3).

### Comparative genomics of the two parental genomes

**Figure 4:**
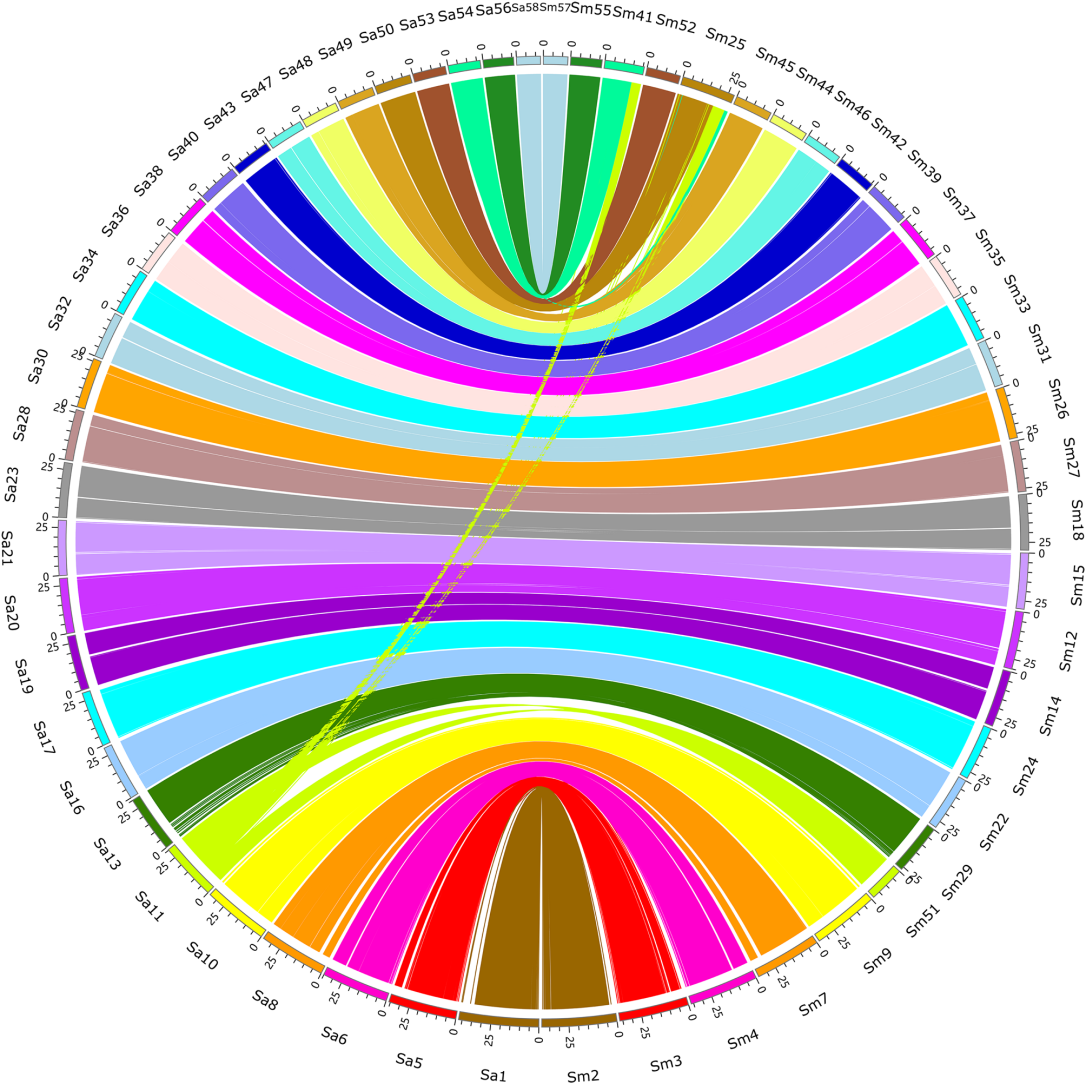
Circos plot demonstrates the high collinearity between homologous chromosomes of *S. asotus* and *S. meridionalis*. Chromosomes of *S. asotus* begin with “Sa”, and chromosomes of *S. meridionalis* begin with “Sm.” Each arc line represents a collinear region.

The genomic sequences of the two species were further assessed by comparing genomes. First of all, homologous chromosomes of the two genomes were compared mutually, and were compared to the *P. fulvidraco* genome, separately. All three comparisons suggested high collinearity between homologous chromosomes of the three species (Fig. 4; Supplemental Figures S3, S4). Secondly, genome sequences of the two parental species were assembled purely based on the short reads of non-hybrid individuals and were combined and compared with the hybrid genome to identify possible recombination regions between the species (Table 2). As a result, 577,880 and 153,897 contigs with a total size of 973,148,448 and 994,540,331 bps were obtained for *S. asotus* and *S. meridionalis*, respectively. The assembled genomes were larger than the estimated genome sizes, and this may be because the redundant sequences were not removed. Comparison analysis showed that 216,289 contigs, accounting for 29.56% of the total 731,777 contigs of the concatenated genome, can be mapped to the hybrid genome. The total length of the aligned regions reached 1,248,990,190 bps and accounted for about 63.47% of the total length of the concatenated genome. There were 1,190,306 1-to-1 regions between the concatenated genome and the hybrid genome, and the total length accounted for nearly 83.58% of the total aligned regions. More than 95% of the 1-to-1 alignment regions account for more than 97% of the aligned bases harboring hits from the same species, that is, bases from *S. asotus* in the concatenated genome were mapped to the sequences of *S. asotus* in the hybrid genome, and the same was true for *S. meridionalis* (Table 2).

**Table 2:** Comparison of the concatenated genomes from the two non-hybrid parental species (query) with that of the hybrid individual (reference).

Indexes	Reference	Query
**[Sequences]**		
TotalSeqs	58	731,777
AlignedSeqs	58 (100.00%)	216,289 (29.56%)
UnalignedSeqs	0 (0.00%)	515,488 (70.44%)
**[Bases]**		
TotalBases (bp)	1,492,906,581	1,967,688,779
AlignedBases (bp)	1,223,495,960 (81.95%)	1,248,990,190 (63.47%)
UnalignedBases (bp)	269,410,621 (18.05%)	718,698,589 (36.53%)
**[Alignments]**		
1-to-1	1,190,306	1,190,306
TotalLength (bp)	1,034,785,460	1,043,886,885
Query and references are from same species	1,136,368 (95.47%)	1,136,368 (95.47%)
TotalLength (bp)	1,003,907,196 (97.02%)	1,012,974,344 (97.04%)
Query and references are from different species	53,938 (4.53%)	53,938 (4.53%)
TotalLength (bp)	30,878,264 (2.98%)	30,912,541 (2.96%)

The percentages included in the brackets denotes the ratio of the number account for total number.

### Annotations of the two parental genomes

About 30% of the genome of each of the two species was composed of REs, and this was also true for the related species *P. fulvidraco* (Table 3; Supplemental Table S3). DNA transposons was the most represented RE and accounted for 20.87%, 21.63%, and 13.89% of the total genome of *S. asotus*, *S. meridionalis*, and *P. fulvidraco*, respectively. The second most represented was LTR, which accounted for about 5% of the whole genome for each of the species, followed by LINE, MITE, and SINE (Table 3; Supplemental Table S3). The total count for each RE type between *S. asotus*, *S. meridionalis*, and *P. fulvidraco* showed that their distribution across homologous chromosome pairs was similar, and suggested a conserved nature (Supplemental Figures S3, S4). Gene model predictions identified a total of 22,894 and 23,273 protein coding genes in the *S. asotus* and *S. meridionalis* genomes, respectively. The total number of coding genes are approximately the same as in the related species *P. fulvidraco*, which has 24,552. Moreover, the distributions of gene length, CDS length, exon length, intron length, which are around 15,000 bp, 1700 bp, 170 bp, and 1500 bp, together with the number of exons and introns, are similar between the three species (Supplemental Figure S5).

**Table 3:** The total number (count), total length (in bp) and its ratio account for the whole genome for major repeat elements reside in each of the three genomes including *S. asotus*, *S. meridionalis*, and *P. fulvidraco*.

Repeat type	*S. asotus*			*S. meridionalis*			*P. fulvidraco*		
	Count	Length	Ratio (%)	Count	Length	Ratio (%)	Count	Length	Ratio (%)
DNA	762,853	155,546,032	20.87	846,064	162,052,381	21.63	586,979	101,608,093	13.89
LINE	29,569	11,801,944	1.59	31,734	11,404,113	1.51	50,327	21,467,256	2.92
LTR	138,885	35,143,804	4.73	136,144	38,186,769	5.1	200,761	52,771,222	7.21
MITE	50,948	7,622,133	1.02	40,549	5,694,588	0.76	24,452	3,951,009	0.54
SINE	340	72,850	0.01	842	176,919	0.02	20,757	2,949,092	0.40
Total	1,017,620	221,703,042	29.80	1,084,986	227,510,949	30.39	963,008	202,943,142	27.74

## Discussion

The data size generated in the study is much larger than former genome projects of related species like *P. fulvidraco* and *Glyptosternum maculatum*^25,42^. We supposed this situation may result in the high quality of assembly. The contig N50 length of the two parental genome reached 6.75 Mb and 9.78 Mb, and this was much longer than that of *P. fulvidraco* and *G. maculatum*, which were 1.1 Mb and 993.67 kb, respectively^25,42^. However, the contig N50 length of a recently reported *S. meridionalis* genome reached 13.19 Mb may suggest the superiority of the Nanopore sequencing technology^43^. We suppose that the contig N50 length is one thing, and the correctness of assembly should be more important. The good collinearity between *P. fulvidraco* and the two parental species may suggest the correctness of the assembly. The two haploids residing in the somatic cells of the hybrid individual may reduce the complexity of assembly and also increase the continuousness. The high mapping rates of short genomic reads from non-hybrid individuals of the two parental species, together with the high performance of BUSCO assessments, and the relatively high continuousness suggest the high quality of the two divided genomes. However, the extent to which “genome shock” and somatic recombination influence the hybrid genome is unknown.

In fact, the possible “genome shock” may be the consequence of the activation of REs^5^. Plenty of active REs may cause many insertions and deletions in the hybrid genome. If we mapped the genomic short reads of the parental individuals back to the hybrid assembly, the insert sizes may deviate from the expected length, which is, the insert sequence length during the library construction. However, we scrutinized the mapping results of the libraries with the insert lengths less than 1 kb, and the insert size distributions are in accordance with the expected length of the two species (Fig. 3). Thus, if there were insertions and deletions that resulted from hybridization of the two parental species, they should be rare. If the REs were activated and inserted into new chromosomal locations, the total number and their distributions may be different between species. However, no obvious differences were detected when we checked the total number and the distribution of different RE types across each of the genomes (Table 3, Supplemental Table S3; Fig. 4, Supplemental Figures S3, S4). Furthermore, the expansion of REs may increase the possibility of non-allelic homologous recombination (NAHR), which may destroy the collinearity of homologous chromosomes^44^. In contrast, high collinearity was detected between the homologous chromosomes of *S. asotus*, *S. meridionalis*, and *P. fulvidraco* (Fig. 4, Supplemental Figures S3, S4).

Comparison of the concatenated genome from the two parental genomes assembled purely based on non-hybrid short reads with the hybrid genome showed that almost all of the mappings were expected. Sequences from the same species were closer. However, there were 53,938 regions accounting for 4.53% of the total regions, and the total length of these regions reached 30,912,541 bps accounted for 2.96% of the total aligned regions, harbored sequences from different species (Table 2). Among these, for each of the aligned sequences from the concatenated genome, a total of 14,706 regions overlapped with other alignments harboring sequences from the same species, and sequences of the overlapped regions from the same species are closer for 11,427 regions. For the other 3502 regions, sequences of the two overlapped alignments were extracted and realigned using clustalW, and the source species are difficult to determine in many cases (Supplemental File S1). If using overlapped regions of the sequences from the concatenated genome as reference, and a mismatch from any of the two genomes divided from the hybrid assembly scores 1 and a gap scores 2, a total of 2101 cases may have resulted from recombination between species. A total of 1817 cases occurred between homologous chromosomes, and 284 account for about 13.5% of the cases that occurred between nonhomologous chromosomes. However, the concatenated genome is not from the two direct parent individuals of the hybrid, so we cannot exclude the possibility of recombination between individuals of the same species. The remaining 39,232 aligned regions did not show any other overlapped alignments between sequences from the same species, and the sequences from the divided hybrid assembly may have also resulted from recombination between species. Pearson’s product–moment correlation showed that the number of these recombination cases may be related to the chromosome length (r = 0.43762, p value = 0.000592). Thus, the recombination may occur stochastically along the chromosome. For the sake of conservation, we provided a version that masked all of the regions that have 1-to-1 aligned regions between different species as Xs for the divided genomes from the hybrid assembly.

## Conclusions

In the present study, two chromosomal-level parental genomes were obtained by sequencing the F1 hybrid genome. The high continuousness and completeness, and the high collinearity with other species, together with the high level of consistency with the assembly using only short-reads from the non-hybrid parents, suggest the high quality of the assembly. Hybridization may significantly reduce the heterozygosity for each parental species since only the haploid genome is present in the somatic cells of the F1 hybrid. Our analysis suggested that if there are insertions, deletions, and recombination that may be consequences of hybridization “genome shock”, they should be rare in the F1 genome. This strategy may significantly improve the assembly quality of highly heterozygous species. Polyploidy fish is a major component in teleost but is notorious for its complex genome, which always result in poor assembly, even if it is based on long reads sequencing technology^9,45^. We suppose that the hybridization strategy of using appropriately selected species may significantly improve the situation since distant hybridization between different fish species seems to be feasible for some tribes like cyprinids^46,47^.

## Supplementary Information

Supplementary Information 1.

Supplementary Information 2.

Supplementary Information 3.

Supplementary Information 4.

Supplementary Information 5.

Supplementary Information 6.

Supplementary Information 7.

Supplementary Information 8.

Supplementary Information 9.

Supplementary Information 10.

## Data Availability

Raw reads generated in the present study are deposit in the NCBI SRA database under the Accession no. PRJNA644951. The genome sequences and gene model files are available on Figshare under the https://doi.org/10.6084/m9.figshare.12961931.v1.

## Code availability

No specific code was used in this work.
