# Supplementary material for: Sequencing an F1 hybrid of Silurus asotus and S. meridionalis enabled the assembly of high-quality parental genomes
Source: Sci Rep. 2021 Jul 5;11:13797. doi: 10.1038/s41598-021-93257-x (PMC8257616; doi:10.1038/s41598-021-93257-x)
Supplement: Supplementary file 1 — Supplementary Information 1. [file 41598_2021_93257_MOESM1_ESM.docx]

**Supplemental Figure S1** Seventeen-mer distribution deduced using genomic short reads from the hybrid individual.

**Supplemental Figure S2** BUSCO assessment results of the de novo assembled genome for the hybrid individual.

**Supplemental Figure S3** Circos plot demonstrates the high collinearity between homologous chromosomes of *S. asotus* and *P. fulvidraco*. Chromosomes of *S. asotus* begin with “Sa,” and chromosomes of *P. fulvidraco* begin with “Pf.” Each arc line represents a collinear region. The distributions of repeat element numbers across each chromosome are also shown. Each track represents a major type, and from the outer to inner are DNA, LINE, LTR, MITE, and SINE. The statistics are based on a 100kb window with no overlap.

**Supplemental Figure S4** Circos plot demonstrates the high collinearity between homologous chromosomes of *S. meridionalis* and *P. fulvidraco*. Chromosomes of *S. meridionalis* begin with “Sm,” and chromosomes of *P. fulvidraco* begin with “Pf.” Each arc line represents a collinear region. The distributions of repeat element numbers across each chromosome are also shown. Each track represents a major type, and from the outer to inner are DNA, LINE, LTR, MITE, and SINE. The statistics are based on a 100kb window with no overlap.

**Supplemental Figure S5** A-F denote the distribution of gene length, CDS length, exon length, intron length, exon number per gene, and intron number per gene for the three relative species *S. asotus, S. meridionalis, and P. fulvidraco*.

**File legends**

**Supplemental File S1** Realignments for the regions in which the identity between sequences from the same species is lower than that between different species, which were identified by mummer using the concatenated genome as query and the de novo assembly for the hybrid individual as reference. All 3,534 overlapping cases are shown and the target regions from the reference genome are extended 100bps up- and downstream; lines1-5 are the sequences of the overlapped regions that mapped to both of the parental sequences, sequences mapped to the first species, the mapped sequences of the first species, sequences mapped to the second species, and the mapped sequences of the second species for each case.

**Supplemental Table S1** The mapping rate of genomic short reads downloaded from NCBI SRA against the divided genomes from the hybrid genome for each of the two species *S. asotus* and *S. meridionalis*.

**Supplemental Table S2** Raw data generated from the *S. asotus* **×** *S. meridionalis* hybrid genome in the present study.

**Supplemental Table S3** Details of major repeat elements reside in each of the three genomes. Each of the total count, total length (in bp) and its ratio account for the whole genome sequences are presented.
