# Supplementary figures and images for: Sequencing an F1 hybrid of Silurus asotus and S. meridionalis enabled the assembly of high-quality parental genomes
Source: Sci Rep. 2021 Jul 5;11:13797. doi: 10.1038/s41598-021-93257-x (PMC8257616; doi:10.1038/s41598-021-93257-x)

# 17-mer Depth Distribution Curve

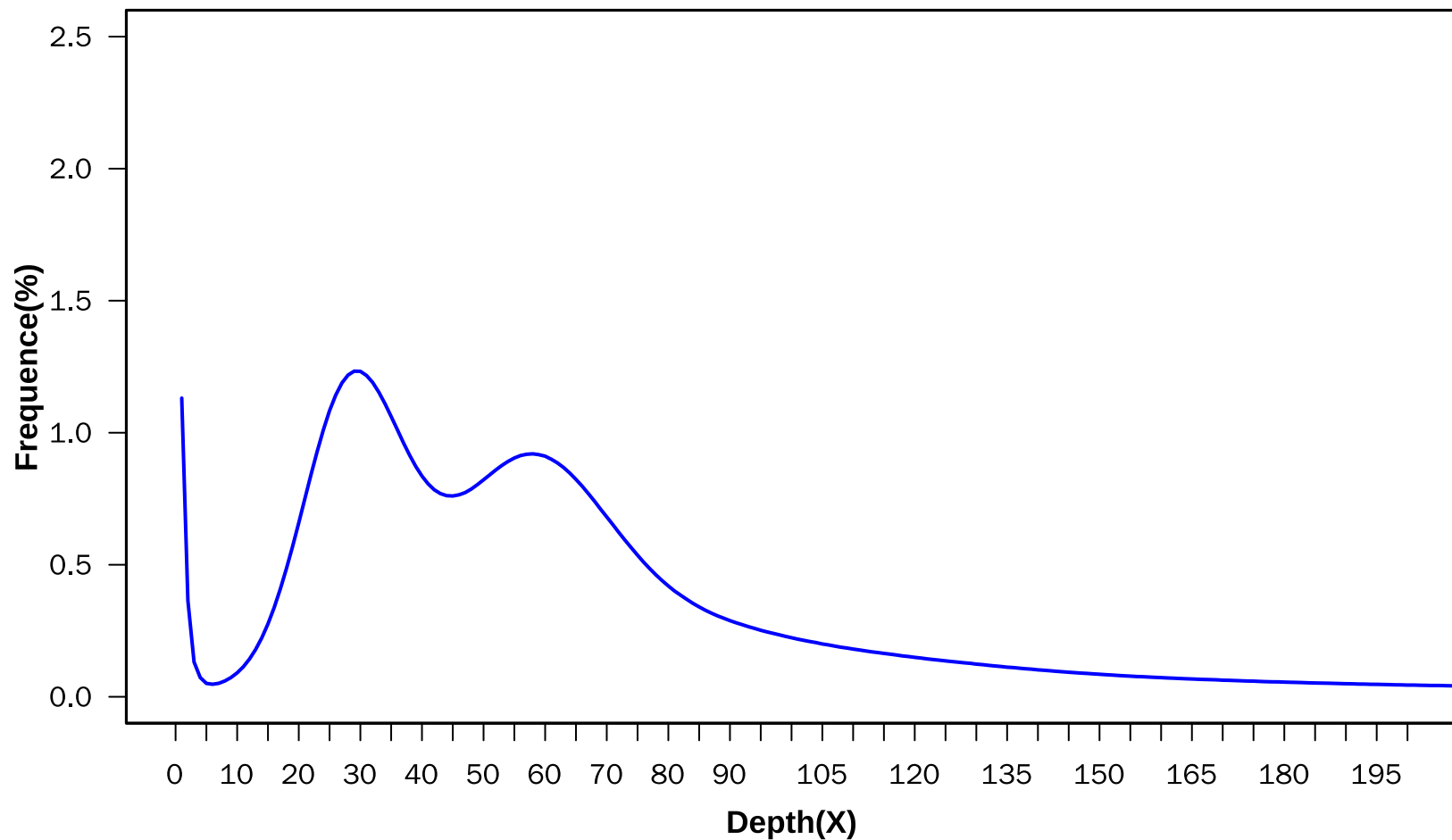

Supplement: Supplementary file 2 — Supplementary Information 2. [file 41598_2021_93257_MOESM2_ESM.pdf]

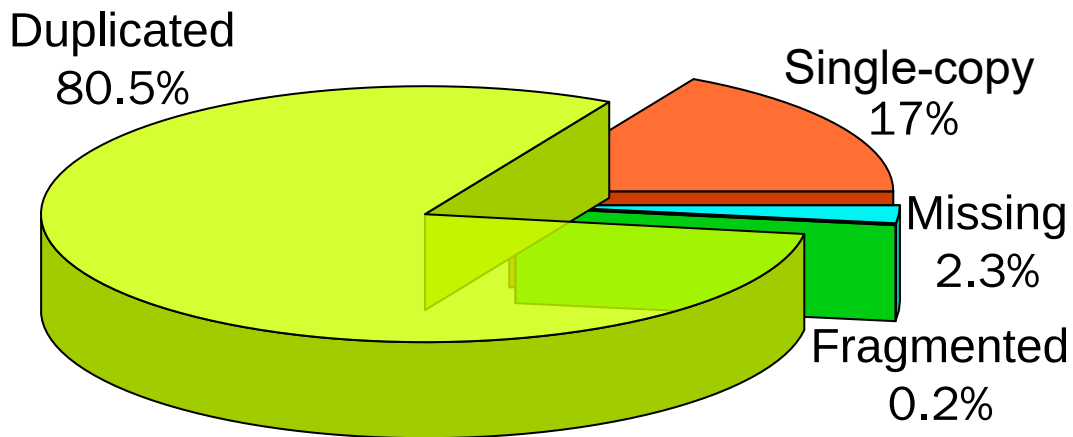

Supplement: Supplementary file 3 — Supplementary Information 3. [file 41598_2021_93257_MOESM3_ESM.pdf]

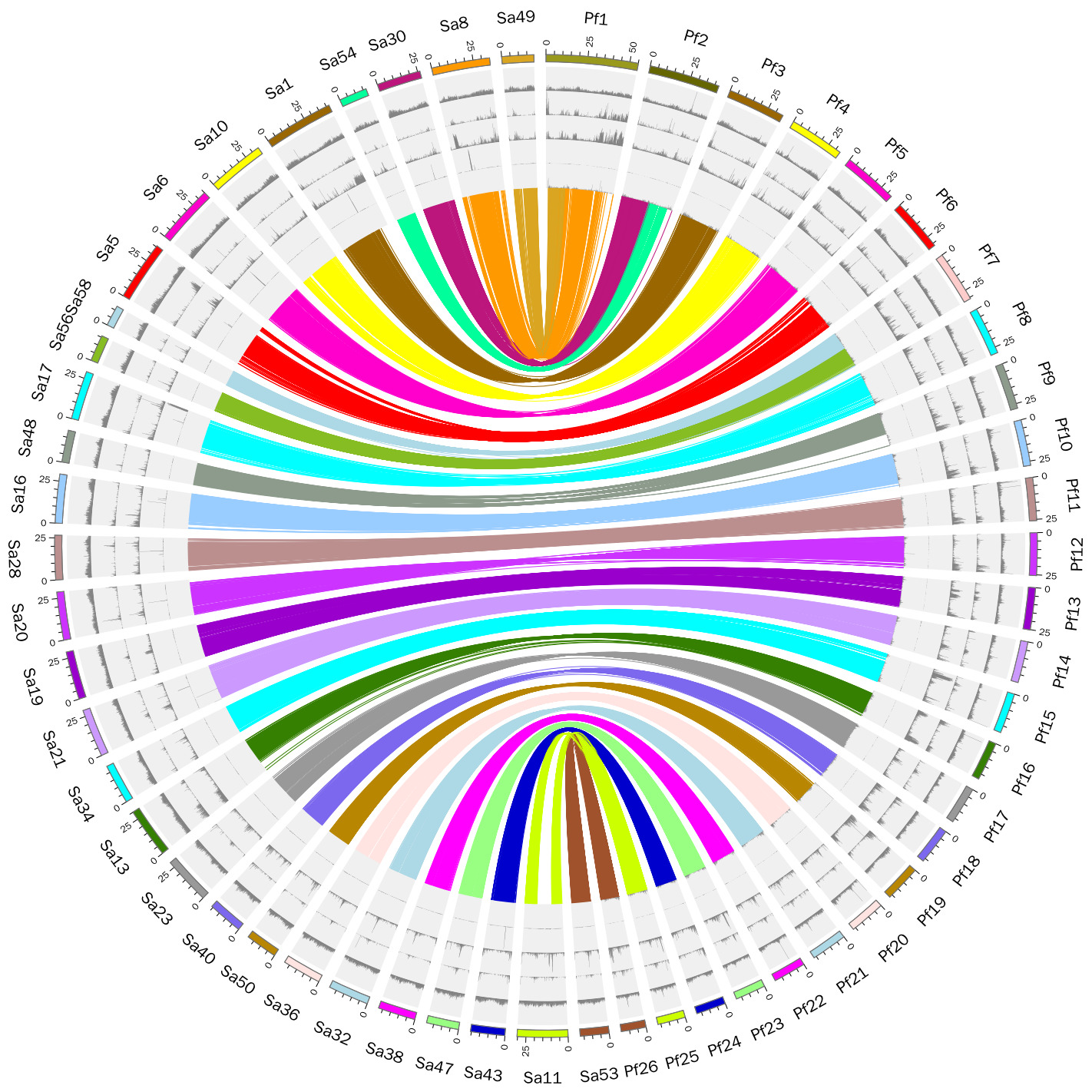

Supplement: Supplementary file 4 — Supplementary Information 4. [file 41598_2021_93257_MOESM4_ESM.pdf]

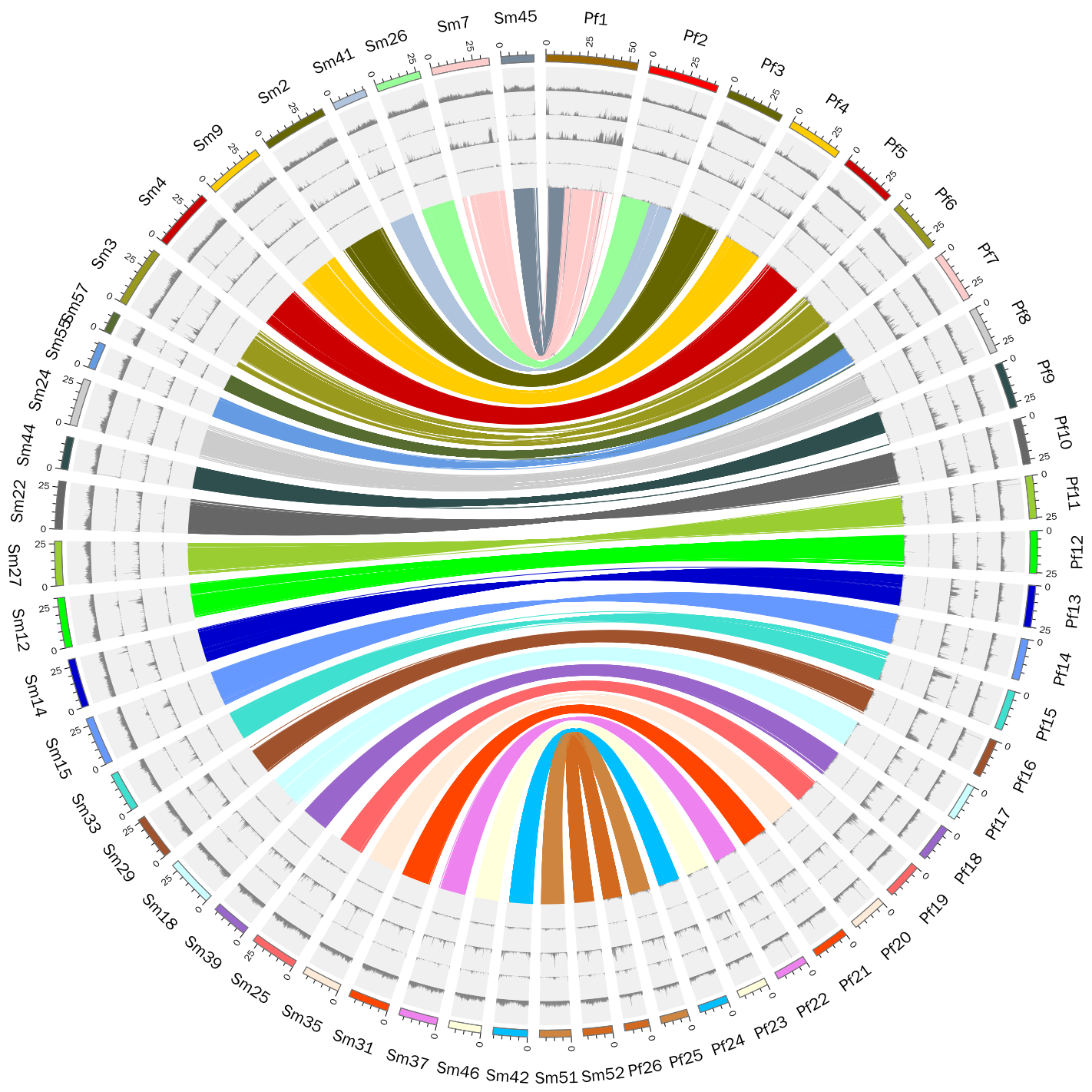

Supplement: Supplementary file 5 — Supplementary Information 5. [file 41598_2021_93257_MOESM5_ESM.pdf]

A

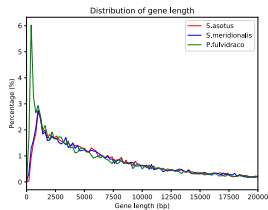

B

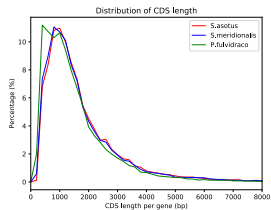

C

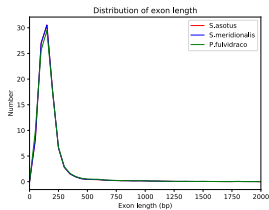

D

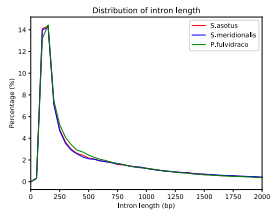

E

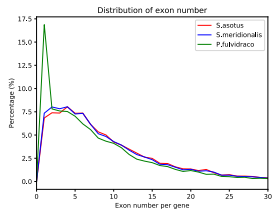

F

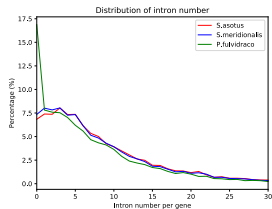

Supplement: Supplementary file 6 — Supplementary Information 6. [file 41598_2021_93257_MOESM6_ESM.pdf]
