# Supplementary material for: Sequencing an F1 hybrid of Silurus asotus and S. meridionalis enabled the assembly of high-quality parental genomes
Source: Sci Rep. 2021 Jul 5;11:13797. doi: 10.1038/s41598-021-93257-x (PMC8257616; doi:10.1038/s41598-021-93257-x)
Supplement: Supplementary file 7 — Supplementary Information 7. [file 41598_2021_93257_MOESM7_ESM.docx]

| *S. asotus* | Mapping rate | *S. meridionalis* | Mapping rate |
| --- | --- | --- | --- |
| SRR7168768 | 93.21% | SRR7188276 | 98.64% |
| SRR7168769 | 92.71% | SRR7188277 | 98.12% |
| SRR7168770 | 98.85% | SRR7188278 | 98.67% |
| SRR7168771 | 92.46% | SRR7188279 | 99.61% |
| SRR7168772 | 96.18% | SRR7188280 | 99.50% |
| SRR7168773 | 95.48% | SRR7188281 | 99.55% |
| SRR7168774 | 93.51% | SRR7188282 | 99.49% |
| SRR7168775 | 96.16% | SRR7188283 | 99.58% |
| SRR7168776 | 98.83% | SRR7188284 | 99.62% |
| SRR7168777 | 96.59% | SRR7188285 | 99.58% |
| SRR7168778 | 96.59% | SRR7188286 | 99.63% |
| SRR7168779 | 96.22% | SRR7188287 | 98.68% |
| SRR7168780 | 97.07% | SRR7188288 | 98.67% |
| SRR7168781 | 97.07% | SRR7188289 | 98.51% |
| SRR7168782 | 96.94% | SRR7188290 | 97.17% |
| SRR7168783 | 98.91% | SRR7188291 | 98.78% |
| SRR7168784 | 98.91% | SRR7188292 | 98.69% |
| SRR7168785 | 98.90% | SRR7188293 | 98.75% |
| SRR7168786 | 98.90% | SRR7188294 | 98.72% |
| - | - | SRR7188295 | 98.75% |
| - | - | SRR7188296 | 98.08% |
| - | - | SRR7188297 | 98.03% |
| - | - | SRR7188298 | 98.48% |
