# Supplementary material for: Sequencing an F1 hybrid of Silurus asotus and S. meridionalis enabled the assembly of high-quality parental genomes
Source: Sci Rep. 2021 Jul 5;11:13797. doi: 10.1038/s41598-021-93257-x (PMC8257616; doi:10.1038/s41598-021-93257-x)
Supplement: Supplementary file 8 — Supplementary Information 8. [file 41598_2021_93257_MOESM8_ESM.docx]

| Sequencing type | application | tissue | platform | Number of raw data sequences | Size of raw data (Gb) |
| --- | --- | --- | --- | --- | --- |
| Genome-Seq | Survey | White muscle | Illumina HiSeq 2000 | 773,198,050 | 115.97 |
|  | *De novo* |  | PacBio Sequel II | 8,675,042 | 126.38 |
|  | Hi-C |  | Illumina HiSeq 2000 | 712,822,844 | 106.92 |
| RNA-Seq | Assist annotation | intestines | Illumina HiSeq 2000 | 57,604,376 | 8.64 |
|  |  | skin |  | 44,003,386 | 6.60 |
|  |  | kidney |  | 42,525,572 | 6.38 |
|  |  | gill |  | 50,303,674 | 7.55 |
|  |  | spleen |  | 69,326,218 | 10.40 |
