# Supplementary material for: Sequencing an F1 hybrid of Silurus asotus and S. meridionalis enabled the assembly of high-quality parental genomes
Source: Sci Rep. 2021 Jul 5;11:13797. doi: 10.1038/s41598-021-93257-x (PMC8257616; doi:10.1038/s41598-021-93257-x)
Supplement: Supplementary file 9 — Supplementary Information 9. [file 41598_2021_93257_MOESM9_ESM.docx]

| Repeat type | | *S. asotus* | | | *S. meridionalis* | | | *P. fulvidraco* | | |
| --- | --- | --- | --- | --- | --- | --- | --- | --- | --- | --- |
|  |  | count | length | ratio | count | length | ratio | count | length | ratio |
| DNA | Total | 762,853 | 155,546,032 | 20.87% | 846,064 | 162,052,381 | 21.63% | 586,979 | 101,608,093 | 13.89% |
|  | CMC-EnSpm | 13,886 | 2,582,823 | 0.35% | 13,877 | 2,345,045 | 0.31% | 34,017 | 4,039,197 | 0.55% |
|  | DTA | 111,429 | 25,133,936 | 3.38% | 142,624 | 30,738,229 | 4.11% | 106,073 | 20,133,600 | 2.75% |
|  | DTC | 269,369 | 53,052,040 | 7.13% | 304,069 | 52,118,764 | 6.96% | 164,461 | 25,943,226 | 3.55% |
|  | DTH | 26,905 | 3,975,275 | 0.53% | 31,795 | 5,454,472 | 0.73% | 18,794 | 2,595,951 | 0.35% |
|  | DTM | 148,500 | 24,040,883 | 3.23% | 182,716 | 30,146,864 | 4.03% | 87,987 | 15,588,198 | 2.13% |
|  | DTT | 31,040 | 8,078,970 | 1.09% | 22,438 | 3,469,742 | 0.46% | 13,841 | 2,056,625 | 0.28% |
|  | Helitron | 43,964 | 8,338,036 | 1.12% | 24,333 | 4,931,990 | 0.66% | 31,333 | 5,235,278 | 0.72% |
|  | IS3EU | 960 | 258,024 | 0.03% | 1,052 | 190,566 | 0.03% | 6,455 | 761,325 | 0.10% |
|  | Maverick | 5,918 | 599,646 | 0.08% | 7,071 | 632,291 | 0.08% | 11,300 | 1,142,979 | 0.16% |
|  | PIF-Harbinger | 3,827 | 693,282 | 0.09% | 4,335 | 745,865 | 0.10% | 9,043 | 1,875,643 | 0.26% |
|  | PiggyBac | 2,863 | 817,040 | 0.11% | 2,654 | 650,029 | 0.09% | 2,214 | 661,300 | 0.09% |
|  | TcMar-ISRm11 | 1,583 | 438,664 | 0.06% | 3,774 | 974,990 | 0.13% | 2,190 | 787,398 | 0.11% |
|  | TcMar-Tc1 | 51,276 | 18,748,517 | 2.52% | 53,524 | 21,036,885 | 2.81% | 39,045 | 11,614,969 | 1.59% |
|  | Zisupton | 1,391 | 307,015 | 0.04% | 842 | 147,910 | 0.02% | 2,829 | 826,593 | 0.11% |
|  | hAT-Ac | 23,983 | 3,855,819 | 0.52% | 26,447 | 4,389,302 | 0.59% | 20,474 | 2,895,118 | 0.40% |
|  | hAT-Tip100 | 917 | 230,326 | 0.03% | 1,230 | 328,054 | 0.04% | 5,031 | 873,350 | 0.12% |
|  | hAT-Charlie | 6,082 | 769,159 | 0.10% | 6,074 | 816,049 | 0.11% | 3,100 | 351,265 | 0.05% |
| LINE | Total | 29,569 | 11,801,944 | 1.59% | 31,734 | 11,404,113 | 1.51% | 50,327 | 21,467,256 | 2.92% |
|  | I | 429 | 133,615 | 0.02% | 593 | 199,553 | 0.03% | 2,012 | 786,110 | 0.11% |
|  | L1 | 2,705 | 1,197,345 | 0.16% | 2,719 | 1,136,469 | 0.15% | 2,979 | 1,351,326 | 0.18% |
|  | L1-Tx1 | 2,790 | 1,048,138 | 0.14% | 2,577 | 913,960 | 0.12% | 3,853 | 1,697,358 | 0.23% |
|  | L2 | 9,885 | 4,443,132 | 0.60% | 9,238 | 4,121,280 | 0.55% | 14,578 | 7,129,827 | 0.97% |
|  | Penelope | 1,044 | 165,627 | 0.02% | 1,170 | 168,338 | 0.02% | 1,699 | 673,056 | 0.09% |
|  | R2-Hero | 223 | 119,609 | 0.02% | 225 | 105,698 | 0.01% | 1,508 | 429,664 | 0.06% |
|  | RTE-BovB | 137 | 28,799 | 0.00% | 65 | 23,383 | 0.00% | 3,588 | 3,324,300 | 0.45% |
|  | Rex-Babar | 12,089 | 45,85328 | 0.62% | 14,716 | 46,36839 | 0.62% | 19,878 | 60,22395 | 0.82% |
| LTR | Total | 138,885 | 35,143,804 | 4.73% | 136,144 | 38,186,769 | 5.1% | 200,761 | 52,771,222 | 7.21% |
|  | DIRS | 6,274 | 2,308,489 | 0.31% | 6,843 | 2,377,112 | 0.32% | 2,120 | 1,218,986 | 0.17% |
|  | ERV1 | 8,247 | 1,105,626 | 0.15% | 9,512 | 1,222,573 | 0.16% | 13,782 | 1,907,133 | 0.26% |
|  | Gypsy | 40,117 | 12,149,554 | 1.63% | 32,009 | 11,903,883 | 1.59% | 46,399 | 16,483,337 | 2.25% |
|  | Ngaro | 1,026 | 115,262 | 0.02% | 1,187 | 128,186 | 0.02% | 5,881 | 628,755 | 0.09% |
|  | Pao | 998 | 492,518 | 0.07% | 1,009 | 529,457 | 0.07% | 1,376 | 819,203 | 0.11% |
| MITE | Total | 50,948 | 7,622,133 | 1.02% | 40,549 | 5,694,588 | 0.76% | 24,452 | 3,951,009 | 0.54% |
|  | DTA | 18,502 | 2,920,096 | 0.39% | 21,694 | 3,324,417 | 0.44% | 13,197 | 2,259,145 | 0.31% |
|  | DTC | 15,002 | 1,975,835 | 0.27% | 11,645 | 1,538,027 | 0.21% | 3,498 | 512,955 | 0.07% |
|  | DTH | 386 | 81,983 | 0.01% | 701 | 98,237 | 0.01% | 4,177 | 577,676 | 0.08% |
|  | DTM | 16,825 | 2,620,365 | 0.35% | 6,453 | 7,199,38 | 0.10% | 3,553 | 596,202 | 0.08% |
| SINE | Total | 340 | 72,850 | 0.01% | 842 | 176,919 | 0.02% | 20,757 | 2,949,092 | 0.40% |
| Satellite | Total | 1,340 | 657,457 | 0.09% | 1,444 | 546,561 | 0.07% | 2,400 | 1,056,863 | 0.14% |
| Simple repeat | Total | 2,389 | 2,520,398 | 0.34% | 3,349 | 3,396,584 | 0.45% | 5,037 | 3,034,257 | 0.41% |
| Total | | 1,017,620 | 221,703,042 | 29.80% | 1,084,986 | 227,510,949 | 30.39% | 963,008 | 202,943,142 | 27.74% |
